# Baicalin suppress the development of polycystic ovary syndrome via regulating the miR-874-3p/FOXO3 and miR-144/FOXO1 axis

**DOI:** 10.1080/13880209.2023.2208636

**Published:** 2023-06-05

**Authors:** Xiaoyuan Xu, Xiaohua Xu, Xiaoshuang Wang, Ling Shen

**Affiliations:** a Department of Obstetrics and Gynecology, The First Affiliated Hospital of Shantou University Medical College, Shantou, China; b Department of Cardiology, the First People’s Hospital of Jingdezhen, Jingdezhen, China

**Keywords:** Endocrine and metabolic dysfunction, miRNA, *Scutellaria baicalensis*

## Abstract

**Context:**

Polycystic ovary syndrome (PCOS) is a common and complex disease caused by endocrine and metabolic dysfunction in women of reproductive age. Baicalin is reported to ameliorate PCOS.

**Objective:**

This study determines whether baicalin could affect the progression of PCOS.

**Materials and methods:**

To establish an animal model of PCOS, female Sprague-Dawley (SD) rats were subcutaneously injected with dehydroepiandrosterone (DHEA, 60 mg/kg) for 20 days. Next, normal and PCOS mice were divided into 3 groups: control, PCOS, PCOS + Baicalin (20 mg/kg) groups. In addition, the levels of microRNA-874-3p (miR-874-3p) and microRNA-144 (miR-144) in ovarian tissues were assessed by reverse transcription-quantitative polymerase chain reaction (RT-qPCR).

**Results:**

Compared to the PCOS group, baicalin treatment significantly declined free testosterone (33.71 pg/mL vs. 56.05 pg/mL) and luteinizing hormone (LH; 3971.73 pg/mL vs. 5201.50 pg/mL) levels in rats with PCOS. Additionally, compared to the control group, 100 μM baicalin lessened miR-874-3p and miR-144 levels in human ovarian granulosa cells (KGN cells) by 36.87% and 32.57%, respectively. Furthermore, forkhead box O (FOXO) proteins FOXO1 and FOXO3 are the direct targets of miR-144 and miR-874-3p, respectively. Meanwhile, baicalin induced G0-G1 phase arrest (69.56 ± 3.7% at baicalin with 100 μM vs. 51.24 ± 3.2%, control) in KGN cells correlating with decreased p27 Kip1 (FOXO proteins downstream effector gene) expression by 55.5%; however, miR-874-3p or miR-144 overexpression could abolish this effect.

**Conclusions:**

Baicalin could alleviate the symptoms of PCOS *via* regulating miR-874-3p/FOXO3 and miR-144/FOXO1 axis, demonstrating its potential utility in PCOS treatment.

## Introduction

Polycystic ovary syndrome (PCOS) is a common and complex disease caused by endocrine and metabolic dysfunction in women of reproductive age (Patel 2018; Joham et al. 2022). It is characterized by chronic anovulation (abnormal or lost ovulation) and hyperandrogenemia (Azziz 2018; Armanini et al. 2022). In addition, the primary clinical manifestations of PCOS are irregular menstrual cycle, infertility, hirsutism, ovarian polycystic changes, etc. (Zhang, Bao, et al. 2019). Recently, despite the development of approaches (e.g., drug therapy, surgery and assisted fertility therapy, assisted reproductive technology) for PCOS therapy (Naderpoor et al. 2015; Jin and Xie 2018; Khmil et al. 2022; Mendoza et al. 2022), treatment efficacy remains unsatisfactory (Pervaz et al. 2023). Therefore, novel treatment approaches for PCOS needs further study and exploration.

MicroRNA (miRNA) is a class of endogenous small RNAs, about 20–24 nucleotides in length (Tsuzuki and Watanabe 2017; Begum 2022). Previous studies have shown that miRNA is widely present and expressed in ovarian tissues (Deb et al. 2018; Zhang, Xu, et al. 2019; Wang et al. 2022). For example, abnormal expression of miRNAs is observed in follicular membrane cells in PCOS patients (Chen et al. 2019). Moreover, the development of PCOS has a close correlation with miR-874-3p, miR-144 and miR-145 (Xia et al. 2018; Lin et al. 2019; Zong et al. 2019; Shafienia et al. 2022).

Baicalin is a flavonoid glycoside isolated from the dried roots of *Scutellaria baicalensis* Georgi, which is a dicotyledonous Labiaceae plant (Liao et al. 2021). It is reported that baicalin can be used to treat a variety of diseases (Wang et al. 2019; Yu et al. 2019; Wei et al. 2022). For example, Fan et al. (2022) found that baicalin could restore ovarian function in aged mice and improve hydrogen peroxide-induced granulosa cell injury. Chen et al. (2013) showed that baicalin could suppress the growth of ovarian cancer cells. In addition, baicalin was able to ameliorate the progression of PCOS by upregulating AMP-activated protein kinase (AMPK) protein (Wang et al. 2019). Meanwhile, Yu et al. (2019) reported that baicalin effectively reversed the phenomenon of elevated androgen level in the serum of PCOS rats. However, the role of baicalin in PCOS progression remains unclear. Thus, we investigated whether baicalin could affect the progression of PCOS by mediating miRNAs.

## Materials and methods

### Animal study

Animal procedures were approved by the Ethics Committee of The First Affiliated Hospital of Shantou University Medical College (No. SUMC2022-285). NIHG for the Care and Use of Laboratory Animals was followed strictly. Female Sprague-Dawley (SD) rats (21 days old) were purchased from Charles River (Beijing, China). Rats were divided into 3 groups: Control, PCOS, PCOS + Baicalin (20 mg/kg) group, *n* = 5 per group, as described previously (Yi et al. 2020). To establish PCOS model, the rats were subcutaneously injected with DHEA (60 mg/kg; dissolved in 200 μL of sesame oil) for 20 days. Equal volume of sesame oil was injected into rats in the control group. After that, at day 21, rats in the PCOS + Baicalin group were treated orally with 20 mg/kg baicalin every day for another 4 weeks (Yu et al. 2019). Meanwhile, rats in the control group and PCOS group were given orally with equal volume of saline every day for 4 weeks. All rats were euthanized after 4 weeks of treatment with baicalin, and ovarian tissues were collected for subsequent research. In addition, the body weight of each rat was measured before sacrifice.

### Detection of hormone levels

The levels of free testosterone, total testosterone, LH, or follicle-stimulating hormone (FSH) in serum samples from PCOS rats were detected using the Rat F-Testo ELISA Kit (Cat: ELK8742), Rat Testo ELISA Kit (Cat: ELK8314), Rat LH ELISA Kit (Cat: ELK2367) or Rat FSH ELISA Kit (Cat: ELK1315) according to manufacturer’s instructions, respectively. All these kits were provided by ELK Biotechnology (Wuhan, Hubei, China).

### Haematoxylin-eosin (HE) staining

Ovarian tissues were embedded with paraffin, cut into 5 μm sections and then stained with Harris haematoxylin and eosin. Finally, ovarian tissues were examined under a light microscope.

### RT-qPCR

Total RNA was extracted from ovarian tissues or KGN cells using the Trizol reagent. Next, EntiLink™ 1st Strand cDNA Synthesis Kit was used to transcribe RNA into cDNA. Subsequently, qPCR was conducting using EnTurbo™ SYBR Green PCR SuperMix Kit. To calculate the data quantitative measurement of qPCR results, 2^–ΔΔCT^ method was carried out.

### Cell culture and transfection

The human ovarian granulosa cell line (KGN cells) was obtained from Procell Life Science (Wuhan, China). KGN cells were cultured in Dulbecco’s modified Eagle medium Ham’s F12 (DMEM/F12, Thermo Fisher) supplemented with 10% fetal bovine serum (FBS), penicillin and streptomycin under 5% CO_2_ at 37 °C.

MiR-874-3p agomir and miR-144 agomir and miRNA negative control (agomir-ctrl) were obtained from RIBOBIO. KGN cells were transfected with miR-874-3p agomir and miR-144 agomir or agomir-ctrl using Lipofectamine 2000.

### Cell counting kit-8 (CCK-8) assay

KGN cells were seeded into 96-well plates at a density of 5 × 10^3^ cells per well overnight. After that, the CCK-8 reagent (Beyotime) was used to treat KGN cells for 2 h. Subsequently, the absorbance at 450 nm was measured using a microplate reader.

### Terminal deoxynucleotidyl transferase (TdT) dUTP Nick-End labeling (TUNEL) staining

The TUNEL detection apoptosis kit (Millipore, Billerica, MA, USA) was performed to assess cell apoptosis. First, KGN cells were fixed with 4% paraformaldehyde and then treated with TUNEL reagent for 1 h in the darkness. Then, phosphate buffer saline (PBS) was used to wash the cells. Finally, the apoptotic cells were observed under a fluorescence microscope. The nuclei were stained with 4′,6-diamidino2-phenylindole (DAPI).

### Cell proliferation assay

The 5-ethynyl-2′-deoxyuridine (EdU) DNA Proliferation *in vitro* Detection was provided by RIBOBIO. Cells were stained with 50 µM EdU for 1 h, and then stained with Apollo dye reagent. Finally, a fluorescence microscope was used to observe and photograph the EdU-labelled cells.

### Western blot assay

Protein was extracted using the radio immunoprecipitation assay (RIPA) lysis buffer (Aspen Biotechnology, Wuhan, China), and then the bicinchoninic acid (BCA) protein assay kit (Aspen Biotechnology) was used to detect protein concentration. Next, proteins were separated by 10% sodium dodecyl sulphate-polyacrylamide gel electrophoresis (SDS-PAGE) and transferred to polyvinylidene fluoride (PVDF) membranes. Then, membranes were treated with primary antibodies diluted at 1:1,000 including anti-β-actin, anti-B cell lymphoma-2 (Bcl-2), anti-Cleaved caspase 3, anti-FOXO3, anti-FOXO1, anti-p27 Kip1 at 4 °C overnight, followed by incubation with the appropriate secondary antibodies (1:5,000) for 1 h at room temperature. Finally, an enhanced chemiluminescence kit was used for protein exposure. Image J software was applied to quantify the intensity of blots.

### Dual-luciferase reporter assay

The pGL6-miR‐based luciferase reporter plasmids containing wild‐type FOXO3 or mutant FOXO3 3′UTR were designed. Then, plasmids were co-transfected into KGN cells with miR-874-3p agomir using Lipofectamine® 2000. In addition, the pGL6-miR‐based luciferase reporter plasmids containing wild‐type FOXO1 or mutant FOXO1 3′UTR were designed. Then, plasmids were co-transfected into KGN cells with miR-144 agomir using Lipofectamine® 2000. After that, Dual Luciferase Reporter Assay System was performed to evaluate the luciferase activity in cell lysates at 48 h.

### Flow cytometry assay

KGN cells were fixed using 70% ethanol for 24 h. Then, PI/RNase staining buffer was used to treat KGN cells for 30 min at the dark. After that, a BD FACScan flow cytometry was performed to evaluate cell cycle distribution.

### Statistical analysis

The non-parametric analysis was used for the statistical analysis of this study. The experiments were repeated three times and the data were presented as mean ± standard deviation in the present study. The statistical analysis was analysed by GraphPad Prism software (version 7.0, La Jolla, CA, USA). One-way analysis of variance (ANOVA) and Tukey’s test were used to explore the differences among multiple groups. Results were regarded as statistically significant for *p* < 0.05.

## Results

### Baicalin alleviates the symptoms of PCOS in rats

**Figure 1.**
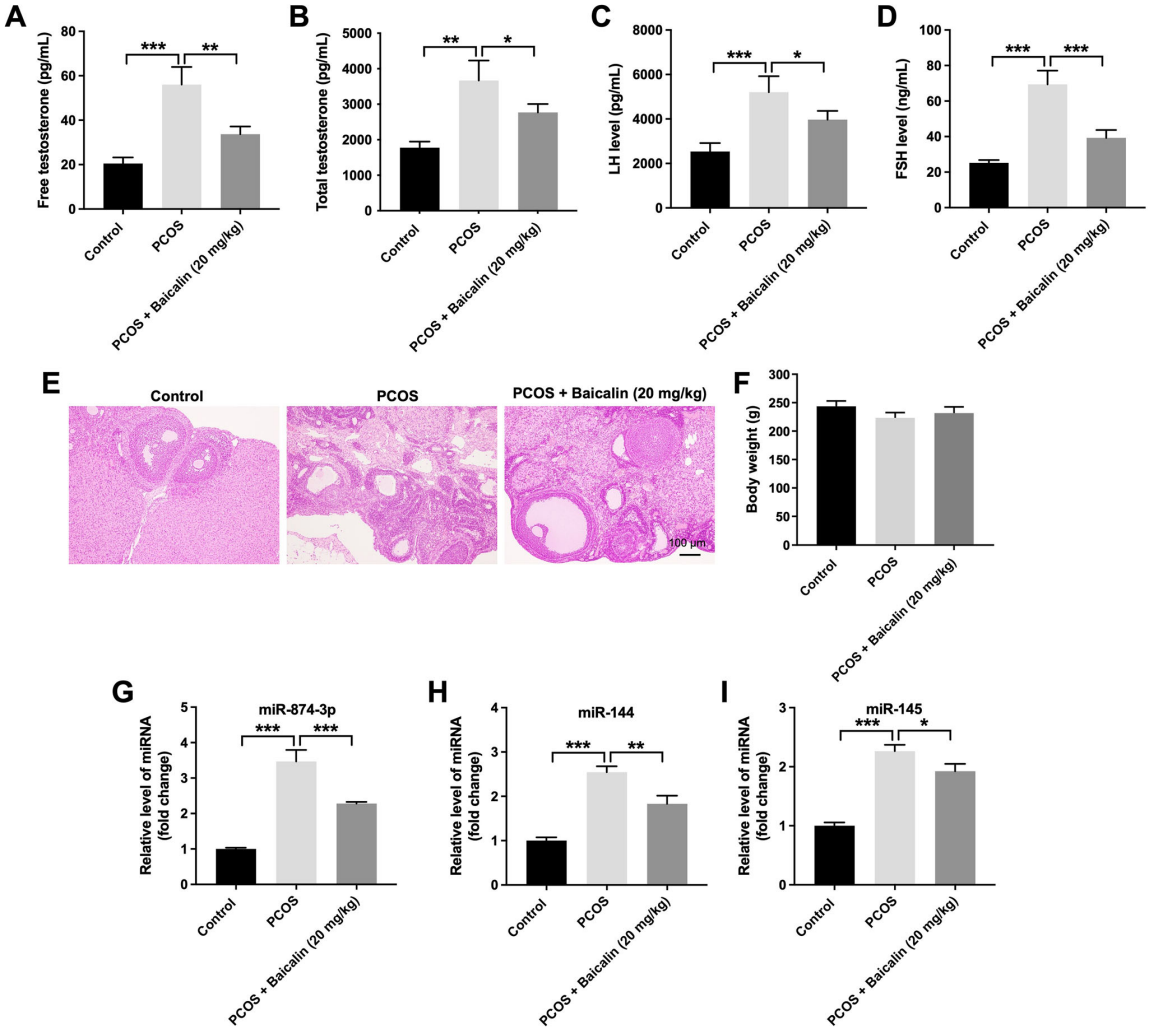
Baicalin alleviates the symptoms of PCOS in rats. (A–D) The levels of free testosterone, total testosterone, LH or FSH in serum samples were detected by ELISA assay. (E) HE staining assay was performed to observe the histopathological changes in ovarian tissues. (F) The body weight of each rat was measured. (G–I) RT-qPCR was performed to evaluate the levels of miR-874-3p, miR-144 and miR-145 in ovarian tissues. *p < 0.05, **p < 0.01, ***p < 0.001; *n* = 3.

A rat model of PCOS was established to explore the role of baicalin in PCOS. The result of ELISA assays illustrated that compared to the control group, the levels of free testosterone, total testosterone, LH or FSH were upregulated in serum samples from PCOS rats, whereas these phenomena were reversed by baicalin treatment (Figure 1(A–D)). In addition, HE staining showed that increased numbers of cystic follicles were observed in the PCOS group, compared to the control group; however, baicalin treatment reversed that effect (Figure 1(E)). Regarding as body weight of SD rats, there was no difference among these three groups (Figure 1(F)). To sum up, baicalin could alleviate the symptoms of PCOS in rats.

We next investigated the relationship between baicalin and miR-874-3p, miR-144 or miR-145 in PCOS. Compared to the control group, the levels of miR-874-3p, miR-144 and miR-145 were remarkably elevated in the ovarian tissues of PCOS rats (Figure 1(G–I)). However, baicalin treatment notably reduced miR-874-3p and miR-144 levels in ovarian tissues of PCOS rats compared to the PCOS group (Figure 1(G,H)). Thus, we focus on exploring the relationship among baicalin, miR-874-3p and miR-144 in the following experiment.

### Baicalin inhibits KGN cell viability and proliferation via downregulating miR-874-3p and miR-144

**Figure 2.**
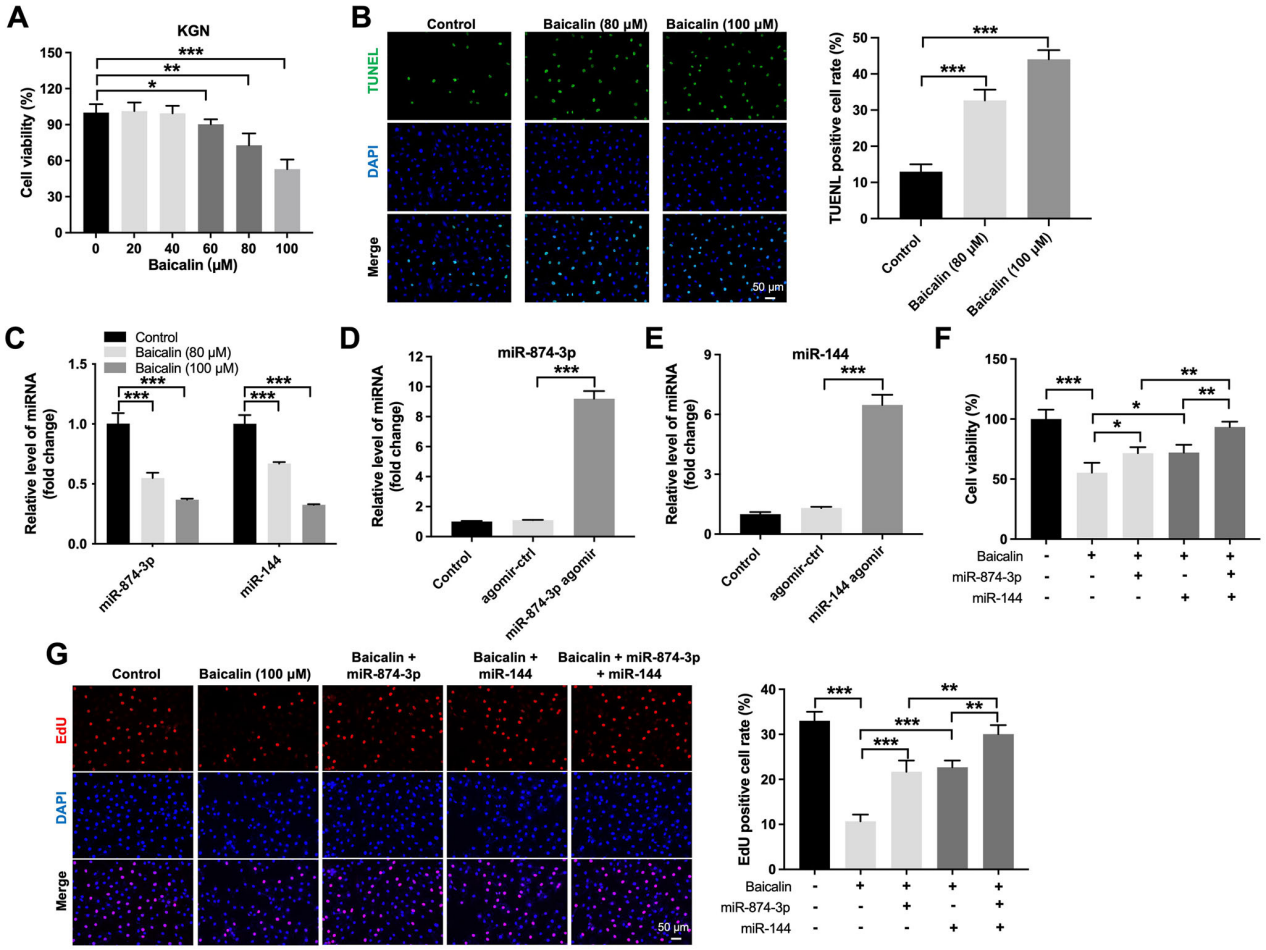
Baicalin inhibits the viability and proliferation of KGN cells *via* downregulating miR-874-3p and miR-144. (A, B) KGN cells were treated with different concentrations of baicalin. CCK-8 and TUNEL were applied to evaluate the viability and apoptosis of KGN cells. (C) RT-qPCR was used to assess the levels of miR-874-3p and miR-144 in baicalin-treated KGN cells. (D) RT-qPCR was used to determine the level of miR-874-3p in KGN cells transfected with miR-874-3p agomir. (E) RT-qPCR was used to detect the level of miR-144 in KGN cells transfected with miR-144 agomir. (F, G) KGN cells were treated with baicalin (100 µM), baicalin + miR-874-3p agomir, baicalin + miR-144 agomir or baicalin + miR-874-3p plus miR-144 agomir. CCK-8 and EdU assays were applied to evaluate the viability and proliferation of KGN cells. *p < 0.05, **p < 0.01, ***p < 0.001; *n* = 3.

Next, we used KGN cells for *in vitro* experiments to explore the mechanisms by which baicalin regulates the development of PCOS. The results of CCK-8 assay implied that 80 and 100 μM baicalin induced about 27% and 47% growth inhibition of KGN cells respectively, suggesting that baicalin could reduce KGN cell viability (Figure 2(A)). Additionally, baicalin significantly triggered cell apoptosis (Figure 2(B)). Moreover, baicalin notably reduced miR-874-3p and miR-144 levels in KGN cells (Figure 2(B,C)). Meanwhile, miR-874-3p agomir or miR-144 agomir obviously increased miR-874-3p or miR-144 level in KGN cells, respectively (Figure 2(D,E)).

**Figure 3.**
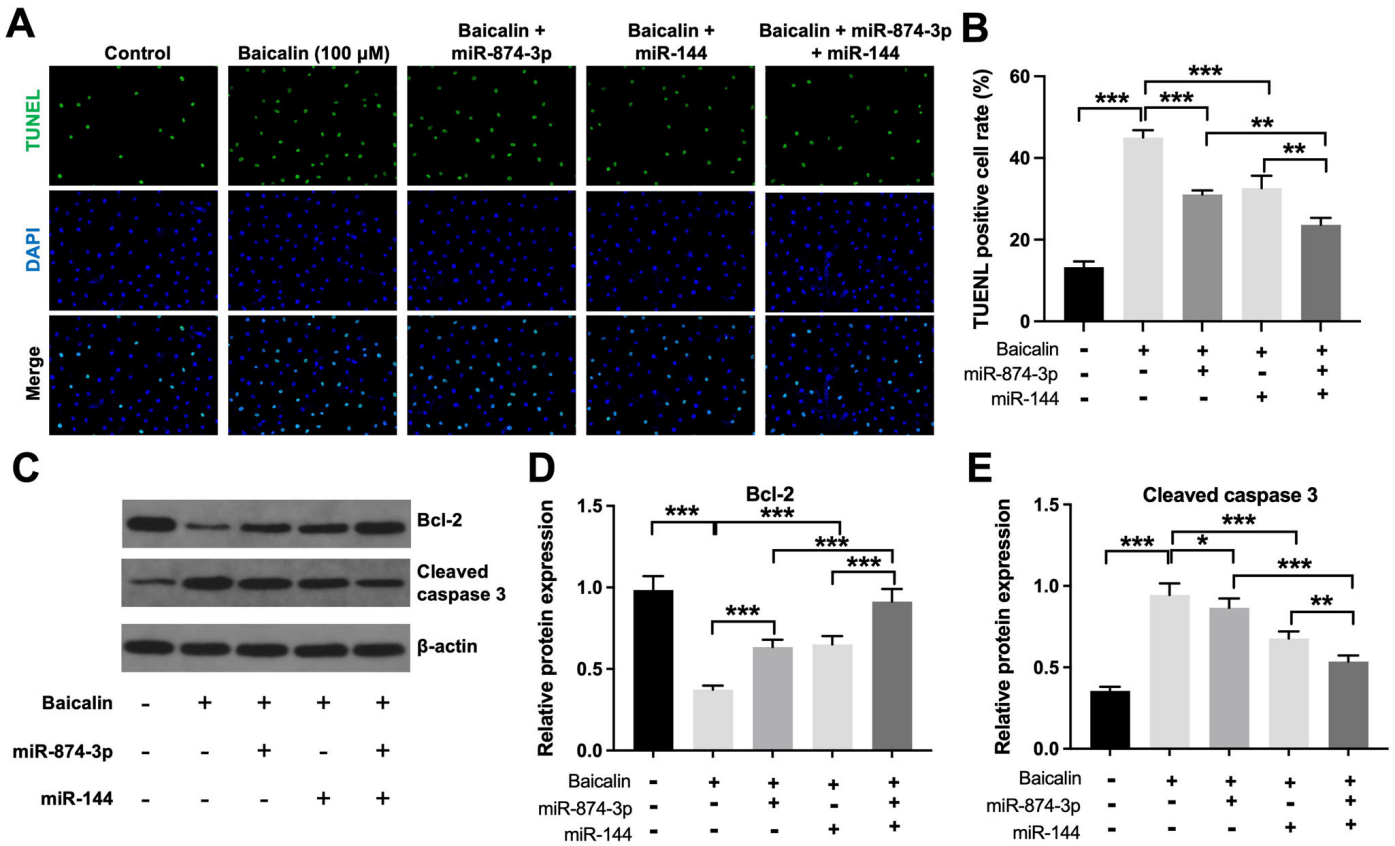
Baicalin induces KGN cell apoptosis *via* downregulating miR-874-3p and miR-144. KGN cells were treated with baicalin (100 µM), baicalin + miR-874-3p agomir, baicalin + miR-144 agomir or baicalin + miR-874-3p plus miR-144 agomir. (A and B) TUNEL was applied to evaluate the apoptosis of KGN cells. (C–E) Western blot assay was applied to evaluate Bcl-2 and cleaved caspase 3 level in KGN cells. *p < 0.05, **p < 0.01, ***p < 0.001; *n* = 3.

Furthermore, the inhibitory effects of baicalin on the viability and proliferation of KGN cells were partly reversed by miR-874-3p agomir or miR-144 agomir (Figure 2(F,G)). Additionally, baicalin significantly triggered KGN cell apoptosis; however, this phenomenon was partly abolished by miR-874-3p agomir or miR-144 agomir (Figure 3(A,B)). Meanwhile, miR-874-3p agomir plus miR-144 agomir treatment remarkably prevented baicalin-induced KGN cell apoptosis, compared to miR-874-3p agomir or miR-144 agomir alone treatment (Figure 3(A,B)). Moreover, baicalin notably reduced the level of Bcl-2 and elevated the expression of cleaved caspase 3 in KGN cells, while these phenomena were partially reversed by miR-874-3p agomir or miR-144 agomir (Figure 3(C–E)). Collectively, baicalin could suppress KGN cell viability and proliferation and trigger cell apoptosis *via* downregulating miR-874-3p and miR-144.

### FOXO3 is a direct target of miR-874-3p and FOXO1 is a direct target of miR-144

**Figure 4.**
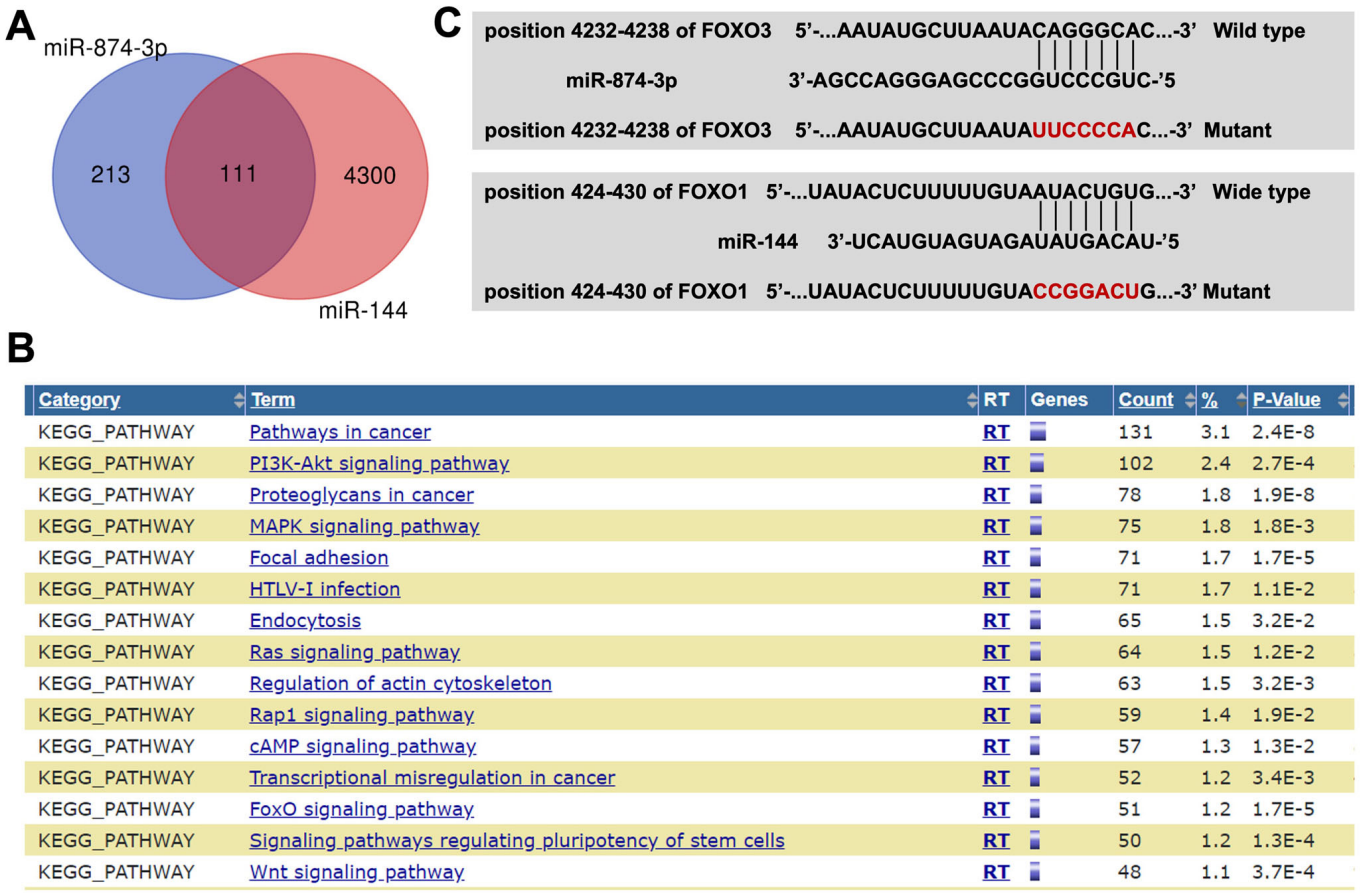
The potential targets of miR-874-3p and miR-144 are explored by using bioinformatics analysis. (A) Venn diagram illustrated that miR-874-3p and miR-144 had 111 common target proteins. (B) The target genes of miR-874-3p and miR-144 were analyzed by KEGG analysis. (C) Sequence alignment of miR-874-3p with the binding sites within the wild type or mutant regions of FOXO3. Sequence alignment of miR-144 with the binding sites within the wild type or mutant regions of FOXO1.

Next, online tools Targetscan and miRWalk were used to explore the potential targets of miR-874-3p and miR-144. The data of Venn diagram illustrated that miR-874-3p and miR-144 had 111 common target proteins (Figure 4(A)). Next, the common target genes of miR-874-3p and miR-144 were analysed by KEGG analysis (Figure 4(B)). The results showed that these target genes were mainly involved in ‘PI3K-Akt signalling’ and ‘FOXO signalling’ pathways (Figure 4(B)). Meanwhile, the data from the Targetscan and miRWalk databases showed that FOXO3 might be a target of miR-874-3p and FOXO1 might be a target of miR-144 (Figure 4(C)), since there two FOXO proteins are closely involve in PI3K-Akt and FOXO signalling pathways.

**Figure 5.**
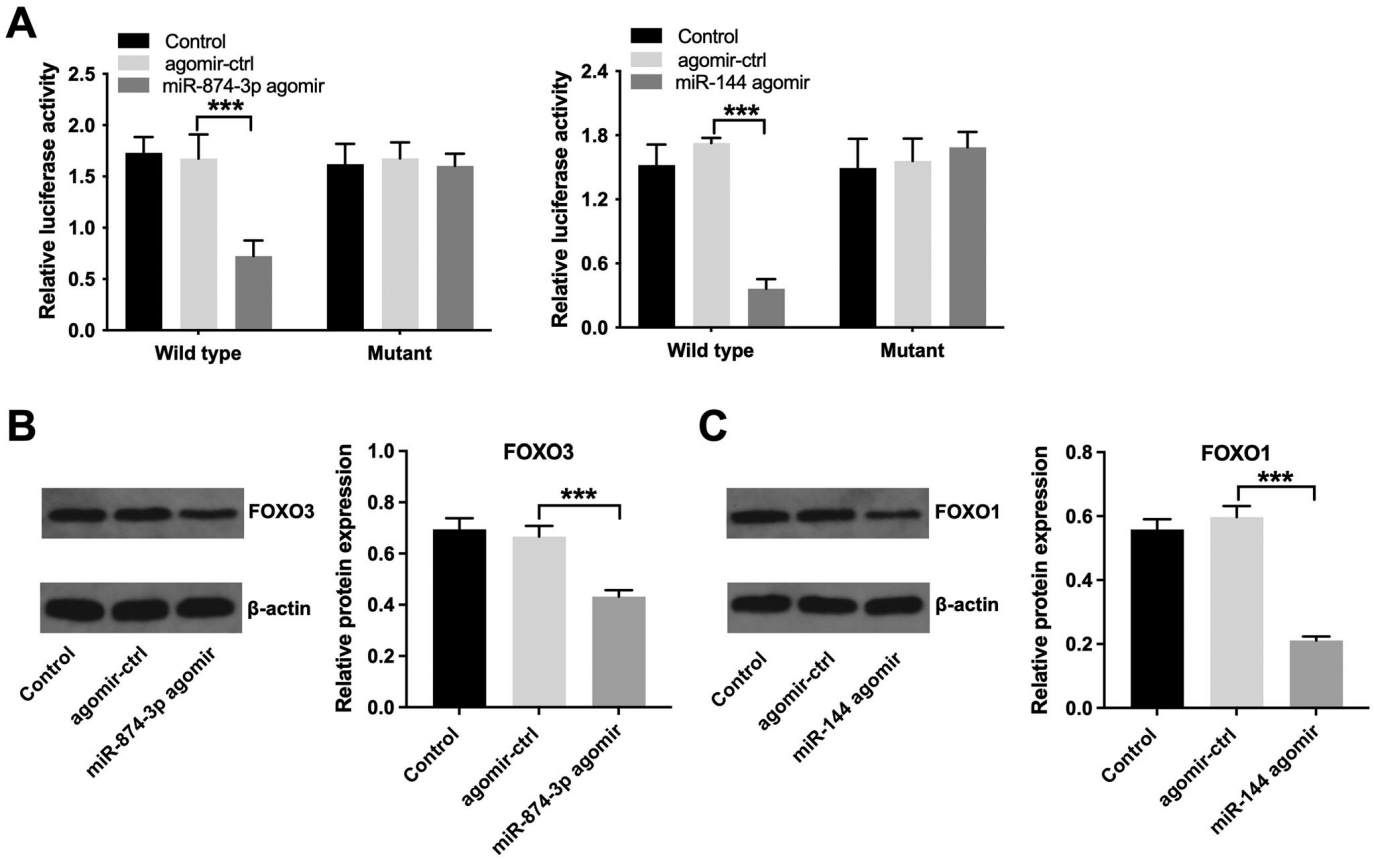
FOXO3 is directly targeted by miR-874-3p and FOXO1 is directly targeted by miR-144. (A) Dual-luciferase reporter assay was applied to verify relationship between potential targets (FOXO3 and FOXO1) and miRNAs (miR-874-3p and miR-144) respectively. (B) Western blot assay was applied to determine FOXO3 level in KGN cells transfected with miR-874-3p agomir. (C) Western blot assay was used to determine FOXO1 level in KGN cells transfected with miR-144 agomir. ***p < 0.001; *n* = 3.

Next, to validate whether FOXO3 or FOXO1 is indeed a target of miR-874-3p and miR-144, respectively, a dual-luciferase reporter assay was performed. As indicated in Figure 5(A), miR-874-3p agomir significantly depleted the luciferase activity of wild type-FOXO3 in KGN cells, and miR-144 agomir depleted the luciferase activity of wild type-FOXO1. Additionally, miR-874-3p agomir notably lessened FOXO3 level and miR-144 agomir weakened FOXO1 level in KGN cells (Figure 5(B,C)). All these results implied that FOXO3 is a direct target of miR-874-3p and FOXO1 is a direct target of miR-144.

### Baicalin suppressed cell cycle progression in KGN cells via downregulating miR-874-3p and miR-144

**Figure 6.**
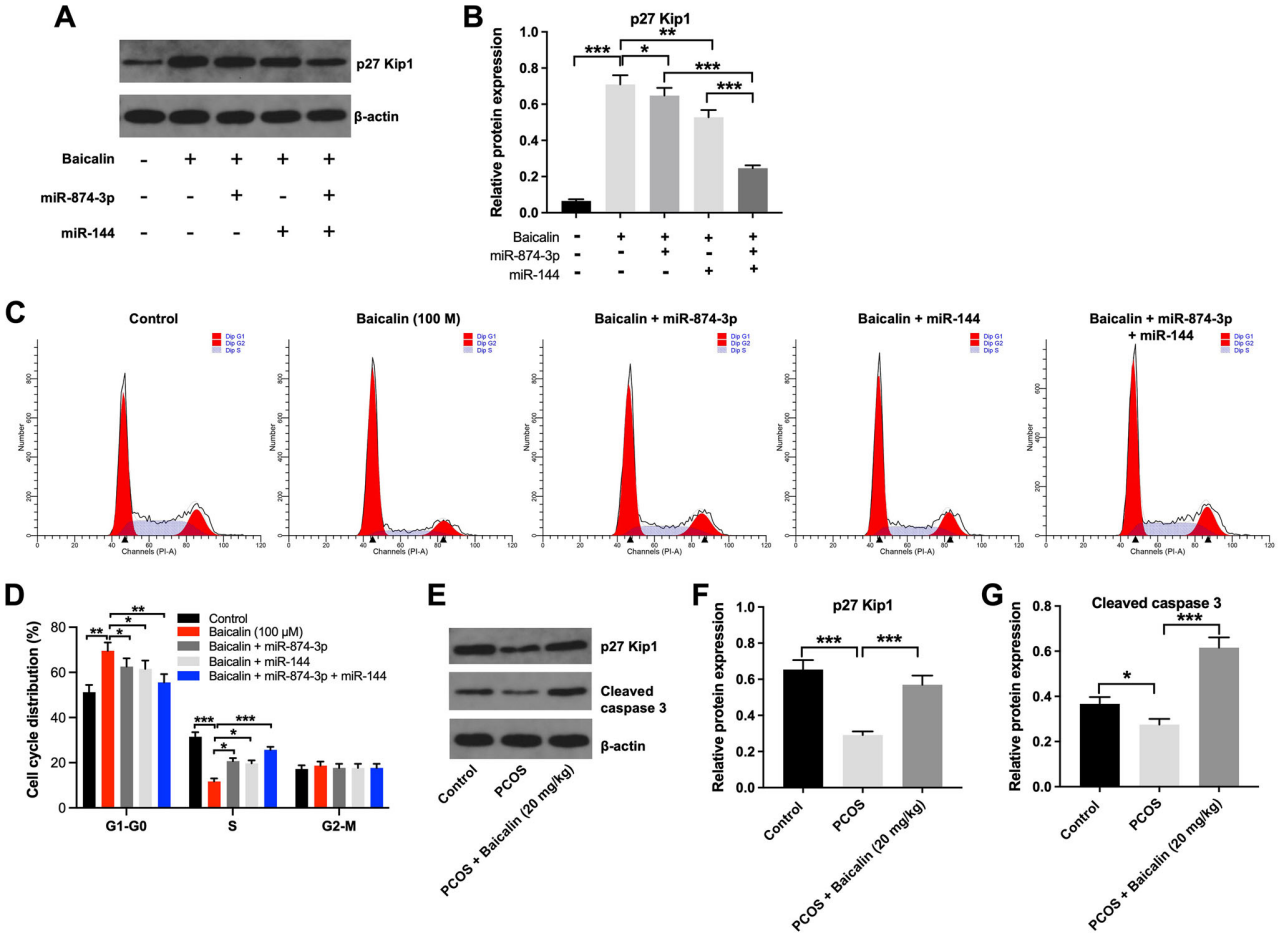
Baicalin induced cell cycle arrest in KGN cells *via* downregulating miR-874-3p and miR-144. (A and B) KGN cells were treated with baicalin (100 µM), baicalin + miR-874-3p agomir, baicalin + miR-144 agomir or baicalin + miR-874-3p plus miR-144 agomir. Western blot assay was applied to evaluate the level of p27 Kip1 in KGN cells. (C and D) Flow cytometry assay was performed to evaluate cell cycle distribution. (E, F and G) Western blot assay was used to assess the levels of p27 Kip1 and cleaved caspase 3 in ovarian tissues from PCOS rats. *p < 0.05, **p < 0.01, ***p < 0.001; *n* = 3.

Evidence has shown that FOXO proteins including FOXO1 and FOXO3 are involved in apoptosis and cell cycle regulation (Morris et al. 2015; Beretta et al. 2019). Thus, we investigated the effect of baicalin on the cell cycle distribution of KGN cells. As shown in Figure 6(A,B), baicalin considerably upregulated the level of p27 Kip1 in KGN cells, while this upregulation was partly reversed by miR-874-3p agomir or miR-144 agomir. As expected, baicalin-induced upregulation of p27 Kip1 in KGN cells was further suppressed by miR-874-3p agomir plus miR-144 agomir, compared to miR-874-3p agomir or miR-144 agomir alone treatment (Figure 6(A,B)). In addition, baicalin elevated the proportion of KGN cells in the G0-G1 phase and decreased the proportions of cells in the S phases; however, these phenomena were reversed by miR-874-3p agomir or miR-144 agomir (Figure 6(C,D)). Moreover, the expression of p27 Kip1 and cleaved caspase 3 was remarkably reduced in ovarian tissues of PCOS rats, compared to the control group (Figure 6(E–G)). However, baicalin treatment was able to reverse these effects (Figure 6(E–G)). Collectively, baicalin could suppress cell cycle progression in KGN cells *via* downregulating miR-874-3p and miR-144.

## Discussion

Emerging evidence has indicated that traditional Chinese medicine exhibited a therapeutic effect on PCOS (Xu et al. 2021; Ji et al. 2022). For example, salidroside is one of the important active ingredients of sedum rosea, which could inhibit dihydrotestosterone (DHT)-induced oxidative stress in KGN cells (Ji et al. 2022). In addition, Liuwei Dihuang Pills could effectively alleviate the symptoms of PCOS in rats by modulating the PI3K/Akt pathway (Qiu et al. 2020). Moreover, baicalin was able to attenuate hyperandrogenism in PCOS rats (Yu et al. 2019). In the current study, we found baicalin treatment significantly reduced the free testosterone, total testosterone, LH and FSH levels in rats with PCOS. In addition, baicalin was able to suppress the viability, proliferation and trigger the apoptosis of KGN cells. These studies suggested that traditional Chinese medicines could relieve the symptoms of PCOS in different ways. Meanwhile, baicalin might be regarded as a therapeutic agent for the treatment of PCOS (Yu et al. 2019; Fan et al. 2022).

It has been shown that miRNAs are differentially expressed in ovarian cells from women with PCOS (McAllister et al. 2019). Zhao et al. (2017) showed that miR-144 level was notably increased in PCOS rats. Wei et al. (2021) found that miR-874-3p level was elevated in granulosa cells collected from patients with PCOS. Additionally, Liu et al. (2022) found that metformin could attenuate the symptoms of PCOS in rats *via* regulating lncRNA/miRNA axis. Therefore, we next explored whether baicalin could suppress the symptoms of PCOS *via* regulating miR-874-3p and miR-144. Our results showed that miR-874-3p and miR-144 levels were obviously elevated in ovarian tissues from PCOS rats. These data showed that miR-874-3p and miR-144 levels were dysregulated in PCOS, which were consistent with previous studies (Zhao et al. 2017; Wei et al. 2021). Baicalin notably weakened miR-874-3p and miR-144 levels in ovarian tissues of PCOS rats. Significantly, baicalin suppressed KGN cell viability and proliferation; however, overexpression of miR-874-3p or miR-144 notably reversed these phenomena. Collectively, baicalin could suppress the proliferation of ovarian granulosa cells *via* downregulating miR-874-3p and miR-144.

Next, online bioinformatics tools were used to identify the potential targets of miR-874-3p and miR-144. We found that FOXO3 might be a target of miR-874-3p and FOXO1 might be a target of miR-144. FOXO1 and FOXO3 are the members of the FOXO protein family (Murtaza et al. 2017). FOXO proteins could reduce cell growth and trigger cell cycle arrest in various cell types (Abid et al. 2005; Kuscu and Celik-Ozenci 2015; Martins et al. 2016). Our results showed that baicalin significantly induced cell cycle arrest in KGN cells as characterized by an accompanying increase in the level of cyclin-dependent kinase inhibitor p27; however, overexpression of miR-874-3p or miR-144 was able to reverse these effects. Moreover, baicalin markedly upregulated p27 level in ovarian tissues of PCOS rats. Collectively, baicalin could suppress cell cycle progression in ovarian granulosa cells through downregulating miR-874-3p and miR-144.

Indeed, there are some limitations in this research. A previous study showed that miR-21 could regulate cell proliferation and apoptosis in PCOS granulosa cells *via* targeting TLR8 (Yu Y et al. 2021). Li et al. showed that miR-1224-5p was able to attenuate the symptoms of PCOS mice through inhibiting the activation of NLRP3 inflammasome *via* targeting FOXO1 (Li et al. 2021). Thus, whether baicalin could affect the progression of PCOS by influencing other miRNAs (e.g. miR-21 and miR-1224-5p) needs to be investigated.

## Conclusions

We found that baicalin could alleviate the symptoms of PCOS *in vitro* and *in vivo via* regulating miR-874-3p/FOXO3 or miR-144/FOXO1 axis. This study showed that baicalin can be exploited as a promising candidate drug to prevent the progression of PCOS.

## Data Availability

The datasets used and/or analyzed during the current study are available from the corresponding author on reasonable request.
